# Management of spontaneous abdominal wall hematomas: a narrative review with a focus on CTA-negative endovascular cases

**DOI:** 10.1186/s42155-025-00647-7

**Published:** 2026-01-15

**Authors:** Erbil Arik, Efe Soydemir, Baris Yer, Onur Taydas, Omer Faruk Topaloglu, Mustafa Ozdemir, Volkan Tasci, Mehmet Halil Ozturk, Bulent Arslan

**Affiliations:** 1 Department of Radiology, Faculty of Medicine, Marmara University, Istanbul, Turkey; 2 Department of Radiology, Faculty of Medicine, Sakarya University, Sakarya, Turkey; 3 Department of Interventional Radiology, Rush University Medical Center, Chicago, IL USA

**Keywords:** Abdominal wall hematoma, Embolization, Iliopsoas muscle, Rectus sheath

## Abstract

**Background:**

Spontaneous abdominal wall hematomas (AWH), typically involving the iliopsoas or rectus sheath, are most often seen in elderly or anticoagulated patients. Their nonspecific presentation can mimic other acute abdominal conditions, delaying diagnosis and management. Computed tomography angiography (CTA), particularly when performed in a multiphasic manner, including venous and delayed phases, provides high sensitivity and specificity in detecting active bleeding. Although subtle arterial bleeding may not always be detectable on arterial-phase imaging alone, this can potentially result in false-negative findings. Treatment options include conservative management, endovascular embolization, and surgical intervention. Identification of the bleeding source on CTA guides targeted embolization during digital subtraction angiography (DSA). In cases where CTA fails to identify the bleeding source, DSA is employed for further assessment and potential embolization. There is no standardized approach in the literature for planning DSA in patients with negative active bleeding signs on preprocedural CTA. This narrative review discusses the clinical presentation, pathophysiology, imaging characteristics, and endovascular treatment options for AWH, with particular emphasis on our procedural approach in patients with negative preprocedural CTA findings.

**Methods:**

We conducted a literature search in PubMed and Google Scholar from inception to December 2024, including studies on spontaneous AWH treated with endovascular embolization. Traumatic hematomas and cases managed exclusively with conservative or surgical methods were excluded. Data from four publications (two systematic reviews and two retrospective studies, totaling 460 patients) were synthesized, and our institutional approach to managing CTA-negative AWH was also summarized.

**Results:**

A total of 460 patients were identified across 4 publications, including 2 systematic reviews (accounting for 408 patients) and 2 retrospective studies. Technical success rates were 100%. In retrospective studies, clinical success rates ranged from 77 to 100%, whereas in two systematic reviews, the reported rates were 56.3% to 89.5% and 93.1%, respectively. Bleeding detection rates were 47% to 82% for CTA and 79% to 85% for DSA. Targeted arteries for embolization were reported, in order of frequency, as follows: lumbar artery, iliolumbar artery, and deep circumflex iliac artery for posterior AWH and deep inferior epigastric artery for anterior AWH.

**Conclusion:**

Endovascular embolization is an effective and safe treatment for spontaneous AWH. DSA remains essential for localization and embolization. In cases with negative CTA, catheterization of arteries anatomically supplying the hematoma is recommended for both diagnostic and therapeutic purposes. Our stepwise, experience-based protocol for CTA-negative cases offers a practical roadmap for interventional radiologists performing embolization in both anterior and posterior AWH.

**Graphical Abstract:**

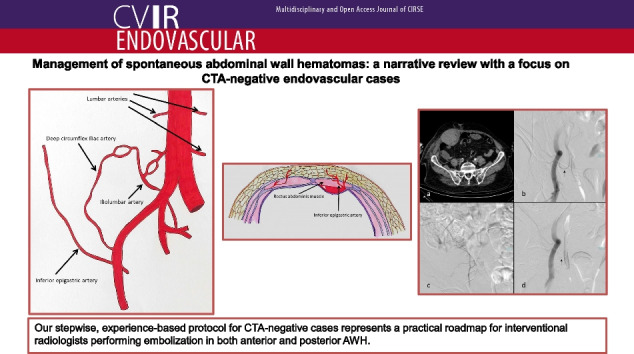

**Supplementary Information:**

The online version contains supplementary material available at 10.1186/s42155-025-00647-7.

## Introduction

Spontaneous hematomas within the iliopsoas muscle and rectus sheath constitute a significant clinical condition leading to emergency department presentations. Clinically, these hematomas may mimic peritonitis and other acute abdominal pathologies. Abdominal wall hematomas (AWH) are commonly classified as posterior AWH (PAWH), localized in the iliopsoas region, and anterior AWH (AAWH), occurring within the rectus sheath. Spontaneous AWH, unlike iatrogenic or traumatic bleeding, primarily affects elderly individuals and patients with coagulation disorders or those receiving antiplatelet or anticoagulant therapy. Throughout this article, the term “AWH” will refer specifically to spontaneous cases, unless otherwise specified.

To date, no prior study or review has outlined a procedural strategy for cases in which pre-procedural imaging fails to demonstrate an active bleeding focus. In the final section of this review, we present our proposed approach for managing such cases.

Clinical presentation of AWH ranges from abdominal pain or a palpable mass to signs of hemorrhagic shock, depending on hematoma severity. Ultrasound (US) represents an important first-line modality in the evaluation of AAWH, not only for confirming the diagnosis but also for excluding other potential etiologies such as intra-abdominal or visceral pathology. Although US offers approximately 90% sensitivity in detecting AAWH, its diagnostic accuracy may be limited in delineating the exact origin or extent of the hematoma, particularly in large hematomas, where cross-sectional imaging such as computed tomography (CT) is often required for comprehensive assessment [1].

Non-contrast CT, contrast-enhanced CT, and CT angiography (CTA) are the most frequently utilized imaging modalities for AWH diagnosis. Non-contrast CT visualizes the hematoma within the abdominal wall, whereas CTA is preferred to detect active bleeding and helps identify the responsible artery. Treatment options include conservative management, endovascular intervention, and surgery, and hemodynamic stability is a critical determinant of treatment decisions. Endovascular treatment involves digital subtraction angiography (DSA) to localize the bleeding focus and achieve hemostasis via embolization [2].

CTA is recommended to identify the bleeding before embolization. The presence of active extravasation or pseudoaneurysm at the hematoma margins or in adjacent tissues suggests bleeding. However, in some cases, bleeding may not be visualized on CTA due to technical limitations, hypotension, or the intermittent nature of the bleeding, including tamponade by surrounding soft tissues. In such clinical scenarios, DSA may be required to identify the bleeding focus. The relative superiority of CTA and DSA in detecting active bleeding remains controversial in AWH. In contrast, for gastrointestinal bleeding, CTA demonstrates higher sensitivity than DSA at low bleeding rates [3].

There is no standardized approach in the literature for planning DSA and subsequent embolization in patients with negative findings on preprocedural CTA or in cases where a related artery cannot be identified despite positive CTA findings. Furthermore, there is no established data concerning the protocol for empirical embolization if DSA fails to detect active bleeding [4]. Several studies have shown no significant difference in technical or clinical success between empirical and targeted embolization [5, 6]. Therefore, the decision to proceed with empirical embolization should be framed not as a fallback but as a justified therapeutic strategy, based on clinical factors such as hemodynamic instability, ongoing anemia, or failure of conservative treatment.

This review aims to summarize the current literature on the endovascular treatment of AWH, encompassing both AAWH and PAWH, and to describe a proposed procedural strategy for CTA-negative cases that has been applied in our patient cohort to date, with 100% technical and clinical success.

## Definitions, epidemiology, and pathophysiology

AAWH results from rupture of the epigastric vessels or rectus muscle fibers within the anterior sheath. Due to the absence of a posterior sheath below the arcuate line, hematomas in the lower abdominal wall are more likely to cause significant hemorrhage and hemodynamic compromise [2]. AAWH may occur spontaneously or as a result of trauma. Spontaneous AAWH predominantly affects elderly patients with comorbidities. The most common underlying cause is anticoagulant therapy [4]. Hematological disorders predisposing individuals to bleeding diathesis can also contribute to AWH [2]. Although the precise pathophysiology remains unclear, proposed mechanisms include arteriosclerosis, occult vasculopathy, autoimmune microangiopathies, or minor traumas (coughing or sneezing) that may result in small-vessel rupture [7]. The condition is more common in females, with a 2–3:1 female-to-male ratio [4]. Although AAWH is generally considered self-limiting, it may have fatal outcomes. The incidence has been reported as 1.8% in patients presenting with acute abdominal pain [2]. Mortality rates in the literature range from 4 to 25%, highlighting its clinical significance [4].

PAWH presents with abdominal tenderness, hypotension, and, in some cases, sensorineural deficits due to retroperitoneal compression. PAWH is defined as retroperitoneal bleeding occurring without preceding trauma or invasive procedures. It can mimic acute abdominal conditions, which complicates diagnosis and carries a potentially fatal prognosis. It is most commonly observed in elderly patients and those receiving anticoagulant therapy [8, 9]. Among patients on anticoagulants, the incidence of PAWH is approximately 0.1–0.6% [10], with a mortality rate up to 22% [9]. Less common causes include hematological disorders, hemodialysis, and abnormal retroperitoneal formations such as cysts, lipomas, aneurysms, or tumors [11].

It has been suggested that during the COVID-19 pandemic, hyperactive fibrinolysis, a lack of coagulation factors, cytokine overproduction, and increased use of anticoagulant medications for prophylactic or therapeutic purposes may have contributed to a rise in the incidence of abdominal wall hematomas [5, 12, 13].

## Imaging

Plain radiography and US are generally insufficient for diagnosing PAWH, as they often provide nonspecific findings. However, these imaging modalities are crucial in the emergency setting for ruling out other etiologies of the acute abdomen [14]. CT is the primary imaging modality for detecting hematomas. In hypotensive patients with negative findings on plain radiography and US, CT can confirm the diagnosis of PAWH. In contrast, in cases of AAWH, the patient’s history and clinical examination are more informative than in PAWH, and the diagnosis is confirmed via US and CT imaging.

**Fig. 1:**
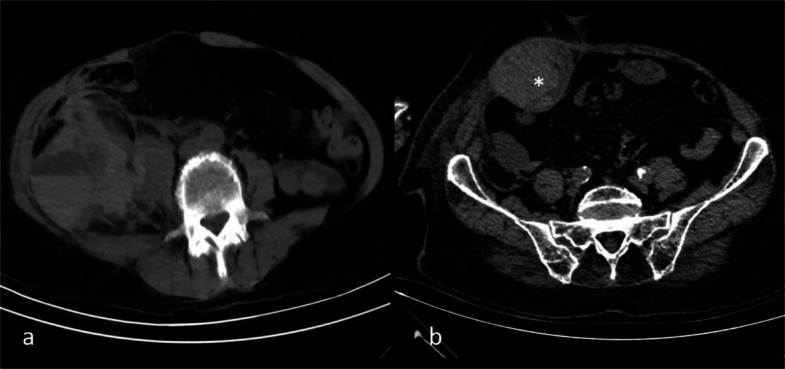
Non-contrast computed tomography showing the hematocrit effect with fluid–fluid levels in a right iliopsoas hematoma (**a**) and the sentinel clot sign, with hyperdense acute clot (asterisk) indicating proximity to active bleeding, in a right rectus sheath hematoma (**b**)

Non-contrast CT provides valuable information regarding the location, size, and age of an AWH. Density measurement is important for determining the age of a hematoma on a CT scan. The relationship between hematoma age and density has been established for intracranial hematomas. Specifically, hematoma density is considered hyperdense compared to brain tissue in the following stages: hyperacute (less than 6 h), acute (6–72 h), and early subacute (3 days to 1 week). During the late subacute phase (1 week to 1 month), the hematoma is isodense, and in the chronic phase (more than 1 month), it becomes hypodense. Initially, hematoma density increases due to the rising concentration of hemoglobin in the leaked blood. Over time, as the blood undergoes liquefaction and lysis, the density decreases [15]. One important imaging feature is the hematocrit effect, which refers to fluid–fluid layering caused by sedimentation of heavier cellular blood components, commonly seen in coagulopathic bleeding (Fig. 1a). The attenuation of clotted blood typically exceeds 60 Hounsfield units (HU), which is higher than that of fresh liquid blood (30–45 HU), enabling estimation of the hematoma’s age based on density. In areas of active bleeding, previously clotted blood may appear hyperdense relative to surrounding components due to earlier coagulation, a finding referred to as the “sentinel clot sign” (Fig. 1b). This imaging feature can help localize the bleeding source [16, 17]. CTA, as the subsequent imaging modality, is particularly valuable in patients with AWH, as it can identify the site of active hemorrhage within the hematoma, from which the likely feeding vessel can be inferred [18]. DSA is the principal imaging modality for guiding embolization in patients undergoing endovascular treatment [2].

## Patient management

The management of AWH depends on the patient’s overall condition, the etiology of the hemorrhage, and its severity. The management approach for PAWH and AAWH is generally similar. Treatment options include conservative management, embolization, and surgery. However, due to limited published data, it remains a controversial issue. In hemodynamically stable patients, conservative management is typically the first-line approach, involving correcting coagulation parameters, fluid resuscitation, and blood transfusion [19, 20]. More aggressive intervention is warranted if the patient does not respond to conservative measures or experiences complications (hemodynamic instability, hematoma expansion, infection/abscess, or abdominal compartment syndrome) [6, 21]. Clinical parameters such as large hematoma size, transfusion requirement of more than 4 units of erythrocytes, and rapid hemoglobin decline, along with imaging evidence of active extravasation on CTA, have been associated with poor response to medical treatment. While current guidelines recommend surgical exploration only in hemodynamically unstable patients who develop abdominal compartment syndrome or neurological complications, the role of endovascular embolization is expanding in life-threatening hemorrhages [22].

Despite detecting hematomas, bleeding may not always be visible in CT scans, making it difficult to pinpoint the exact site of bleeding. In such cases, angiography (DSA) plays a crucial role in identifying the responsible artery and guiding subsequent embolization [6]. According to recent studies, the main indications for DSA and embolization include active extravasation on imaging, large hematoma, and muscular fascia rupture [23]. Based on the studies included in the literature review, although no standardized algorithm has been defined, current evidence suggests that hemodynamic instability should not be considered a contraindication to embolization.

## Literature review and case series on endovascular embolization

### Study selection

We searched the PubMed and Google Scholar databases from inception through December 2024 using the following keywords: “embolization,” “spontaneous,” “hematoma,” “abdominal wall,” “retroperitoneum,” and “rectus sheath.” Searches were limited to English-language publications. We also reviewed the reference lists of included articles to identify any additional relevant studies. Published reviews, case reports, and case series were manually searched.

**Fig. 2:**
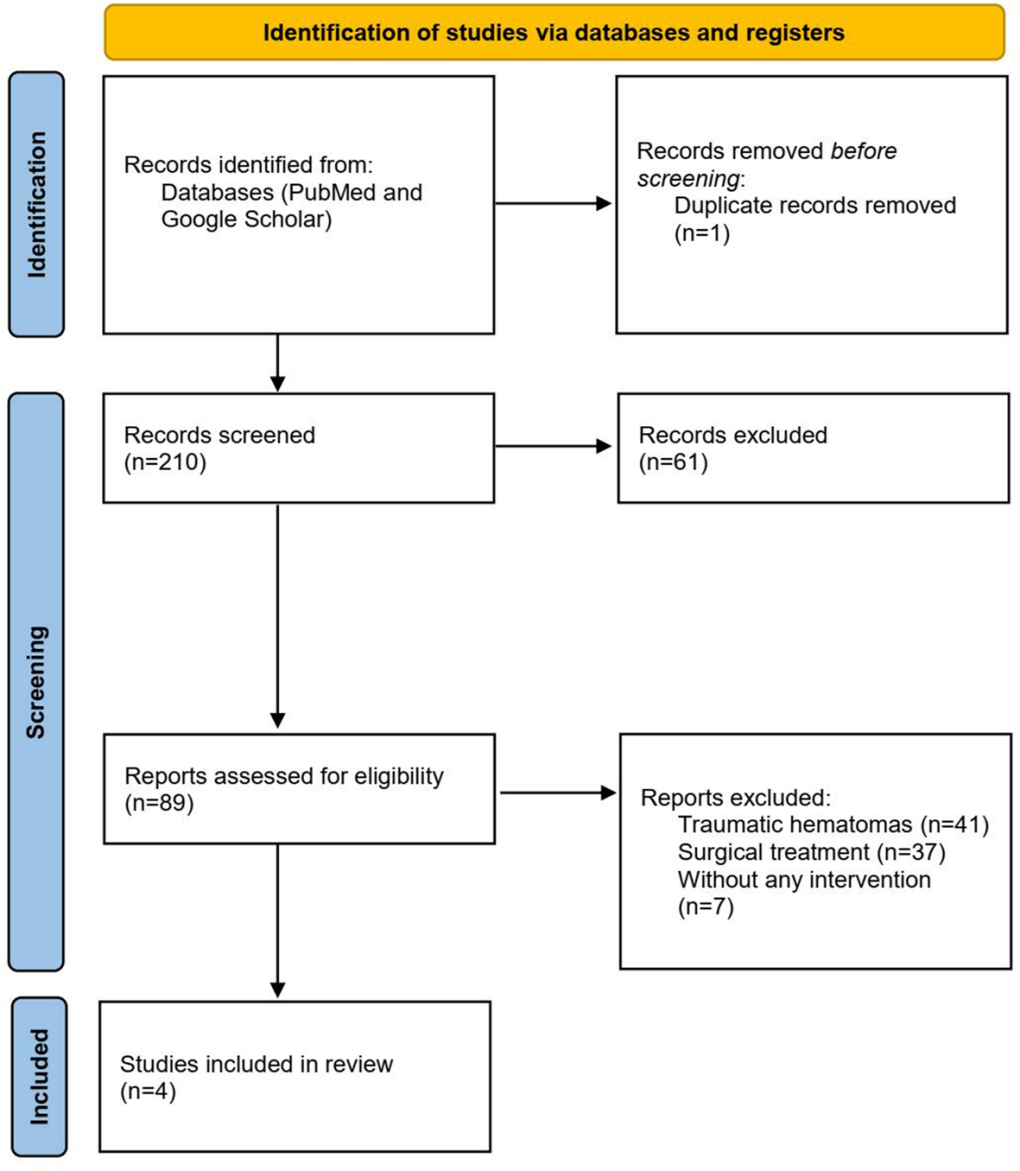
PRISMA flow diagram for study selection

Two authors (AE and TO) independently screened the titles and abstracts of all identified studies. Full-text articles were then reviewed to determine eligibility. Any disagreements were resolved by consensus. Studies were included if they involved patients with spontaneous AWH treated by endovascular embolization. We excluded studies involving traumatic hematomas or those treated only with conservative or surgical methods. Only English-language articles were considered. A total of 460 patients were identified across 4 publications, including 2 systematic reviews (accounting for 408 patients) and 2 retrospective studies (Fig. 2). The source and type of data (retrospective study or systematic review) are presented in Table 1. Although a broad literature search was conducted, this study was designed as a narrative review rather than a systematic review with the aim of summarizing clinical outcomes and incorporating both relevant literature and our own clinical experience with CTA-negative cases.

**Table 1:** Summary of published studies reporting technical and clinical outcomes of endovascular embolization for spontaneous abdominal wall hematoma

Study	Type of the study	Year	Patient number	Location of hematoma^*^	Technical success (%)	Clinical success (%)	Complications (%)
Di Pietro et al. [6]	Retrospective	2022	43	AAWH and PAWH	100	77	2 (1 minor)
López-Martínez et al. [24]	Retrospective	2022	9	PAWH	100	100	0
Touma et al. [25]	Systematic review	2019	267	AAWH and PAWH	_	93.1	0.7 (2 minor, no major)
Tiralongo et al. [5]	Systematic review	2024	141	PAWH	100	56.3 to 89.5%	5.76 (5 minor, no major)

^*^AAWH anterior abdominal wall hematoma, *PAWH* posterior abdominal wall hematoma

## Results

### Technical and clinical outcomes and complication rates

Among the studies included in our review, which evaluated patients undergoing endovascular embolization for spontaneous AWH, the reported technical success rate was 100% in retrospective series [6, 24], and reached 100% in one systematic review [5]. In retrospective studies, clinical success rates ranged from 77 to 100%, whereas in two systematic reviews, the reported rates were 56.3% to 89.5% [5] and 93.1% [25], respectively. The complication rate was reported to range from 0 to 2% in retrospective series and from 0.7% to 5.76% in systematic reviews (Table 1).

In the reviewed studies, technical success was generally defined as the successful catheterization and embolization of the targeted artery, and clinical success was defined as achieving hemorrhage control and eliminating the need for transfusion. Barral et al. investigated factors affecting short-term clinical outcomes in patients who underwent embolization due to soft tissue hematoma. The authors identified a high simplified acute physiology score (calculated based on parameters such as age, heart rate, systolic blood pressure, urine output, selected biochemical values, and chronic comorbidities [26]), along with large hematoma volume, and retroperitoneal localization as predictive factors for poor clinical outcomes [27]. For consistency in reporting, complications described in the included studies were classified as major or minor in accordance with the Society of Interventional Radiology (SIR) reporting standards. Minor complications were defined as events requiring no therapy and without clinical consequences or those necessitating only nominal therapy with no sequelae (including overnight admission for observation only). Major complications were defined as events requiring therapy with short hospitalization (< 48 h), major therapy or unplanned increase in level of care with prolonged hospitalization (> 48 h), as well as those resulting in permanent adverse sequelae or death [28].

When outcomes were examined according to hematoma location, technical success rates were 100% across studies including PAWH or mixed cohorts [5, 6, 24, 25]. Interpretation of clinical success according to hematoma location was more limited. In the PAWH-only series, clinical success rates ranged from 56.3% to 89.5% in a systematic review [5], while a retrospective study reported a 100% clinical success rate [24]. In studies including both AAWH and PAWH, outcomes were not consistently stratified by location, precluding a direct comparison between groups. In these mixed cohorts, clinical success was reported as 93.1% in a systematic review [25] and 77% in a retrospective study [6]. Overall, available data suggest that endovascular embolization is clinically effective for both AAWH and PAWH; however, firm conclusions regarding differential clinical outcomes based on hematoma location cannot be drawn due to study heterogeneity.

### Bleeding detection rates for CTA and DSA, embolized arteries, and embolic agents

**Fig. 3:**
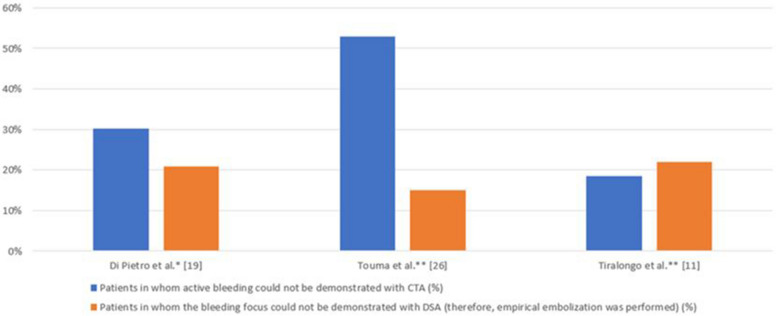
Percentage of patients in whom an active bleeding source could not be identified by CTA or DSA in studies on endovascular embolization for spontaneous abdominal wall hematoma

Several studies have examined the detection rates of active bleeding findings on CTA in patients undergoing embolization for AWH [5, 6, 25]. Among the studies that specified their CTA protocols, Klausenitz et al. [7] used a dual-phase protocol (arterial and venous), whereas Di Pietro et al. [6] employed a triphasic protocol, with pre-contrast imaging followed by arterial, venous, and delayed phases. These rates vary between 47 and 82%. In the same studies, the rate of detecting the bleeding focus during DSA in patients undergoing the procedure for embolization ranges between 79 and 85%. Some studies [6, 25] suggest that DSA is superior in identifying the bleeding focus, whereas others [6, 7] indicate that preprocedural CTA is also compelling. The rates of cases in which an active bleeding focus could not be identified through either CTA or DSA are presented in Fig. 3.

A retrospective study by Dohan et al. found that the sensitivity of CTA and DSA in detecting the bleeding focus was similar. However, the negative predictive value of CTA was low (63%). Among 36 patients, two were CTA-positive but DSA-negative, and four were CTA-negative but DSA-positive. The discrepancy in CTA-positive/DSA-negative cases may reflect a tamponade effect related to the delay between CTA and embolization; however, no data were available to indicate whether the delay between imaging modalities differed from the overall mean interval (39 h; range, 6–312 h). The authors advocated for empirical embolization of feeding arteries in the affected muscular region when DSA failed to identify a bleeding focus [29].

In the studies included in our review, information is provided regarding “which target arteries should be embolized” and “at what point the embolization procedure should be terminated” [5, 6, 24, 25]. In patients with active bleeding identified on pre-procedural CTA, the artery responsible for hemorrhage is targeted. When DSA confirms bleeding (CTA-positive, DSA-positive), targeted embolization is performed; otherwise (CTA-positive, DSA-negative), empirical embolization of the suspected artery is applied. In CTA-negative cases, DSA is primarily used to identify a bleeding focus. When a bleeding focus is demonstrated (DSA-positive), targeted embolization is performed. In the absence of angiographic evidence of bleeding (DSA-negative), empirical embolization is carried out based on hematoma location, most commonly involving the deep inferior epigastric artery (DIEA) for AAWH and the lumbar arteries (LA), iliolumbar artery (ILA), or deep circumflex iliac artery (DCIA) for PAWH. However, neither our review nor the existing literature defines a standardized strategy for bleeding source evaluation in CTA-negative cases or an empirical embolization algorithm when no bleeding focus is identified. Across studies, procedural termination is generally based on angiographic confirmation of technical success, irrespective of the embolic agent used.

There is limited research regarding the arterial origin of bleeding in AWH cases. In these studies, the arteries most frequently responsible for AWH and targeted for embolization have been reported to be the LA, ILA, DCIA for PAWH and DIEA for AAWH (Table 2). Studies in the literature concerning AWH embolization emphasize that the selection of embolic agents may vary based on patient-specific factors and operator experience. Various embolic materials, including Gelfoam®, coils, and liquid embolic agents (N-butyl cyanoacrylate [NBCA], ethylene vinyl alcohol, and particles), as well as combinations of some of these agents, have been used (Table 3) [5, 6, 24, 25].

**Table 2:** Arteries targeted for embolization in studies on endovascular embolization for treating spontaneous AWH

Study	Embolized arteries (AAWH) (in order of frequency)	Embolized arteries (PAWH) (in order of frequency)
Di Pietro et al.^*^ [6]	DIEA	1. LA 2. ILA 3. DCIA
Touma et al.^**^ [25]	DIEA	1. LA 2. DCIA 3. ILA
López-Martínez et al.^*^ [24]		1. LA 1. ILA
Tiralongo et al.^**^ [5]		1. LA 2. ILA 3. DCIA 4. DIEA 5. Intercostal arteries 6. Superior gluteal artery

*Retrospective study, ^**^systematic review

*AWH* abdominal wall hematoma, *AAWH* anterior abdominal wall hematoma, *PAWH* posterior abdominal wall hematoma, *LA* lumbar artery, *ILA* iliolumbar artery, *DCIA* deep circumflex iliac artery, *DIEA* deep inferior epigastric artery

**Table 3:** Embolic agents utilized in studies on endovascular embolization for treating spontaneous AWH

Study	Embolic agents (in order of frequency)
Di Pietro et al.^*^ [6]	1. Coil + gelfoam 2. Gelfoam 3. Coil 4. Liquid agent
Touma et al.^**^ [25]	1. Coil 2. NBCA 3. Gelfoam 4. Coil + gelfoam 5. Coil + microparticle 6. Microparticle
López-Martínez et al.^*^ [24]	1. EVOH 2. Coil
Tiralongo et al.^**^ [5]	1. Gelfoam 2. Coil 3. Coil + gelfoam 4. NBCA 5. NBCA + coil 6. NBCA + gelfoam

*Retrospective study, ^**^systematic review

*AWH* abdominal wall hematoma, *PVA *polyvinyl alcohol, *NBCA* N-butyl cyanoacrylate, *EVOH* ethylene–vinyl alcohol

Severe coagulopathy is widely recognized as a major risk factor for rebleeding, and there is a consensus that liquid embolic agents are well-suited for this patient population due to their effectiveness in reducing the risk of recurrent hemorrhage [24, 30, 31]. Permanent embolic agents have been suggested to be superior to temporary materials such as Gelfoam in preventing rebleeding [29]. Although NBCA was not the first-line embolic material in most of the studies included in this review, it may offer a potential advantage in reducing the risk of recurrent hemorrhage. This is attributed to its ability to achieve complete vascular occlusion even in patients with coagulopathy and to allow control over the degree of polymerization, thereby enabling either proximal or distal embolization as required. However, the risk of non-target embolization, particularly spinal ischemia during lumbar artery embolization, should be carefully considered [7]. Studies in the literature using NBCA as an embolizing agent, primarily involving traumatic AWH cases, reported a 100% technical success rate without any major complications [32–34]. Furthermore, massive blood transfusions administered before embolization may contribute to coagulopathy through mechanisms such as hypothermia, dilutional coagulopathy, platelet dysfunction, fibrinolysis, or hypofibrinogenemia, thus increasing the risk of rebleeding [35]. The underlying mechanism of rebleeding remains uncertain and may be due to incomplete embolization or new bleeding sources. Nevertheless, it has been proposed that expanding hematomas may compromise adjacent vascular structures, leading to the formation of new bleeding foci [7].

### Role and rationality of empirical embolization

Empirical embolization refers to the embolization of a presumed culprit vessel in the absence of angiographic evidence of active extravasation, typically guided by suggestive findings on preprocedural CTA, even when the vessel appears angiographically normal [36].

A retrospective, multicenter study by Tiralongo et al. compared targeted embolization (81%) with empirical embolization (19%) performed without active bleeding findings on DSA in 161 patients with spontaneous AWH [36]. In the empirical embolization group, the target artery was determined based on preprocedural CTA findings. Technical success was reported at 99% and clinical success at 86%, with no significant difference between the two groups. In 30 patients (30/161), multiple arteries were embolized, with the majority (20/30) being PAWH cases, which was attributed to the iliopsoas muscle being perfused by a more significant number of arteries compared to the rectus sheath. There was considerable heterogeneity in embolic agent selection, which was considered to be related to differences in operator preference and experience. Notably, 12% of patients underwent embolization (targeted or empirical) despite the absence of active bleeding on preprocedural CT. However, in this specific subgroup, which constitutes the main focus of our review, the study does not clarify the proportion of patients with positive or negative DSA findings nor does it provide information on how the target artery was selected for empirical embolization in cases with negative DSA [36].

Similarly, a retrospective study by Di Pietro et al. compared targeted and empirical embolization. Among 43 patients (28 with PAWH and 15 with AAWH), active bleeding foci were identified in 31 cases on preprocedural CTA and 34 cases during DSA, leading to targeted embolization. Empirical embolization was performed in nine patients with negative DSA findings for bleeding. The study found that the efficacy and safety of embolization were similar between the two groups. However, as in previous studies, no clear procedural plan was outlined for cases where active bleeding was not detected on either preprocedural CTA or DSA [6].

In a retrospective study, Dohan et al. advocated for empirical embolization of feeding arteries in the affected muscular region when DSA failed to identify a bleeding focus. The authors also recommended acquiring images at the level of the abdominal aorta using a pigtail catheter to avoid missing other bleeding foci [29].

A systematic review by Touma et al. analyzed 63 studies covering 267 patients with spontaneous AWH. The detection rates of the bleeding were 47.7% for preprocedural CTA and 85.6% for DSA. Among the 267 patients, empirical embolization was performed in only 37 without identified bleeding foci. The authors suggested that factors such as hemodynamic status and hematoma size should be considered when deciding on embolization, even without detectable bleeding on CTA. They also reported performing embolization in both hemodynamically stable and unstable patients. The study found that the sensitivity of DSA in identifying the bleeding focus was superior to that of CTA, contrasting with established literature on gastrointestinal bleeding [25]. This discrepancy was attributed to the nature of abdominal wall hemorrhage, which may be more intermittent and initially limited. The recurrence rate was relatively high (9.4%), and there was no correlation between rebleeding and embolic agent selection. Interestingly, the location of recurrent bleeding did not always overlap with the site of the initial bleeding, which was associated with the potential for rebleeding from a different site in cases where the anticoagulant cannot be reversed because of clinical reasons. Only two complications (0.7%) were reported: non-target embolization to a deep femoral artery branch in one and retrograde dissection of the external iliac artery in the other [25].

In a recent systematic review by Tiralongo et al., which included six studies encompassing 116 procedures, empirical embolization was performed in patients without angiographic evidence of active bleeding during DSA in three of the studies (14 out of 72 patients [19.4%]). These empirical embolizations were reported to have a technical success rate of 100%. No significant differences were observed between the targeted and empiric embolization groups in terms of efficacy, safety, or rebleeding rates. The overall rebleeding rate across the six studies was 24.17%, ranging from 10.5% to 31.2%. Thirty-day mortality rates ranged from 5.2% to 43.4% (mean 24.3%), which is consistent with previously published data [5].

Empirical embolization is also utilized in the management of gastrointestinal bleeding and traumatic vascular injuries outside the context of AWH. A meta-analysis by Yu et al., which included 13 studies (12 retrospective and one prospective) on upper gastrointestinal bleeding, reported a clinical success rate of 74.7% (95% CI, 63.1–86.3%). The authors demonstrated that empirical embolization is comparable to targeted embolization in preventing rebleeding and mortality [37]. In trauma settings, empirical embolization of the bilateral internal iliac arteries has similarly been reported to be lifesaving, particularly in hemodynamically unstable patients without an identifiable bleeding source [38].

According to current evidence, empirical embolization in appropriately indicated DSA-negative AWH cases appears to have a life-saving role, considering the high overall mortality associated with these hemorrhages. Despite the absence of angiographically visible extravasation, several reports have shown that empirical embolization yields technical and clinical outcomes comparable to those of targeted embolization, with similar efficacy, safety, and rebleeding rates. When preprocedural CTA demonstrates primary or secondary signs indicating the bleeding source, the corresponding artery can be directly targeted. In contrast, management becomes more challenging when CTA is negative, and to our knowledge, a standardized embolization strategy for these cases has not yet been established in the literature.

In this context, we propose a practical approach based on the anatomical patterns and bleeding tendencies of AWH. For AAWH, we recommend embolization of the DIEA. For PAWH, to minimize the risk of incomplete embolization, we suggest concurrent embolization of the ILA and DCIA, which represent the most likely bleeding sources apart from the LA. Although LAs are most commonly reported as the primary cause of PAWH, empirical embolization of multiple LAs poses considerable technical and safety challenges. The presence of multiple ipsilateral LAs and extensive collateral networks often necessitates embolization at consecutive levels, sometimes up to five arteries (three LAs, ILA, and DCIA) in a single session, which is impractical in the emergency setting. Moreover, this strategy increases the risk of non-target spinal artery embolization, as well as resulting in higher contrast load, radiation exposure, and prolonged procedure time.

## Our clinical practice: how do we perform this procedure?

We describe our institutional approach for endovascular treatment of PAWH and AAWH. This procedure primarily represents the stepwise approach we recommend for CTA-negative cases, encompassing empirical embolization as well. In addition, it provides general technical considerations and practical tips regarding the catheterization and embolization of the aforementioned arteries, which are also applicable to targeted embolization. All procedures are conducted under sterile conditions using an angiography system (Artis Zee; Siemens, Germany), usually under local anesthesia, with sedation or general anesthesia as needed. Equipment includes a 5-Fr sheath (Shoocin; Lepu Medical), a Cobra-2 catheter (Angio-Clean; Taha Biomedical), and a 0.035″ hydrophilic guidewire (Shunmei Medical). Microcatheters (mostly 2.8-Fr Renegade HI-FLO, Boston Scientific and less commonly 2.4-Fr Progreat, Terumo) are chosen based on vessel size and tortuosity. Larger microcatheters improve imaging, but may induce vasospasm in distal vessels.

We first perform diagnostic angiography of the main arteries (aorta, internal and external iliacs) with a Cobra-2 catheter. The angiographic images acquired from the aorta using a diagnostic catheter were not primarily aimed at identifying the bleeding focus, since the Cobra-2 catheter does not allow for adequate contrast opacification. Instead, they were performed to generate a roadmap delineating the aortic and iliac bifurcations to facilitate catheter navigation. Moreover, high-pressure injections performed for aortography may pose a risk of arterial dissection in cases where the catheter tip is not freely positioned within the aortic lumen. In general, for all other injections as well, we prefer to confirm the catheter position by observing blood flush-back or performing gentle manual aspiration after positioning the catheter, in order to prevent this complication. The diagnostic catheter is used for selective imaging, whereas a coaxial microcatheter is employed for selective or superselective angiography. Main artery or selective/superselective angiograms are obtained via manual injection or pump-based protocols. Cone-beam CT (CBCT), obtained via microcatheters using a standardized protocol in our clinic, is valuable for verifying vascular territories [39] (Table 4). If bleeding is visualized or empirical embolization is considered, we selectively advance the microcatheter and proceed. Embolic materials include polyvinyl alcohol particles (Contour; Boston Scientific), and, less commonly, coils (Concerto; Medtronic, and Prestige; Balt Group) or NBCA/lipiodol mixture (Histoacryl; Braun/Iodized oil; Guerbet Laboratories). The choice of embolic agent and its specific properties (including particle size, NBCA/Lipiodol ratio, and coil dimensions) depends on several factors. In our practice, these are selected in accordance with general embolization principles. Given the emergent nature of bleeding, the procedure should be performed efficiently, guided by operator experience. During ILA embolization for PAWH, the potential origin of a spinal branch from the LA must always be considered [40]. To minimize the risk of non-target embolization, we routinely use CBCT, advance the coaxial microcatheter beyond the orifice of any spinal branch, and select relatively large embolic particles (> 300 μm). Particular attention is paid to avoiding reflux or coil protrusion into major vascular structures such as the abdominal aorta, internal or external iliac arteries, or the common femoral artery.

**Table 4:** Our imaging protocol using an injection pump in CBCT

Artery	Frame rate (fps)	Injection rate (ml/sec)	Total amount (ml)	Pressure (PSI)
Abdominal aorta	4	8	24	800
Common iliac artery	4	8	16	300
Internal iliac artery	4	6	12	300
External iliac artery	4	6	12	300
Target artery (for CBCT, via microcatheter)	4	0.5	12	100–300

*CBCT* cone-beam computed tomography

**Fig. 4:**
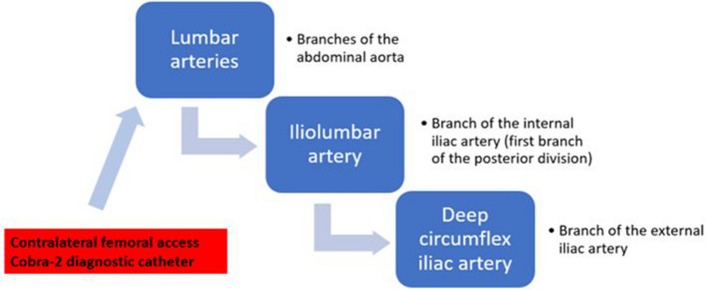
Flowchart for CTA-negative posterior abdominal wall hematoma embolization (CTA: computed tomography angiography)

**Fig. 5:**
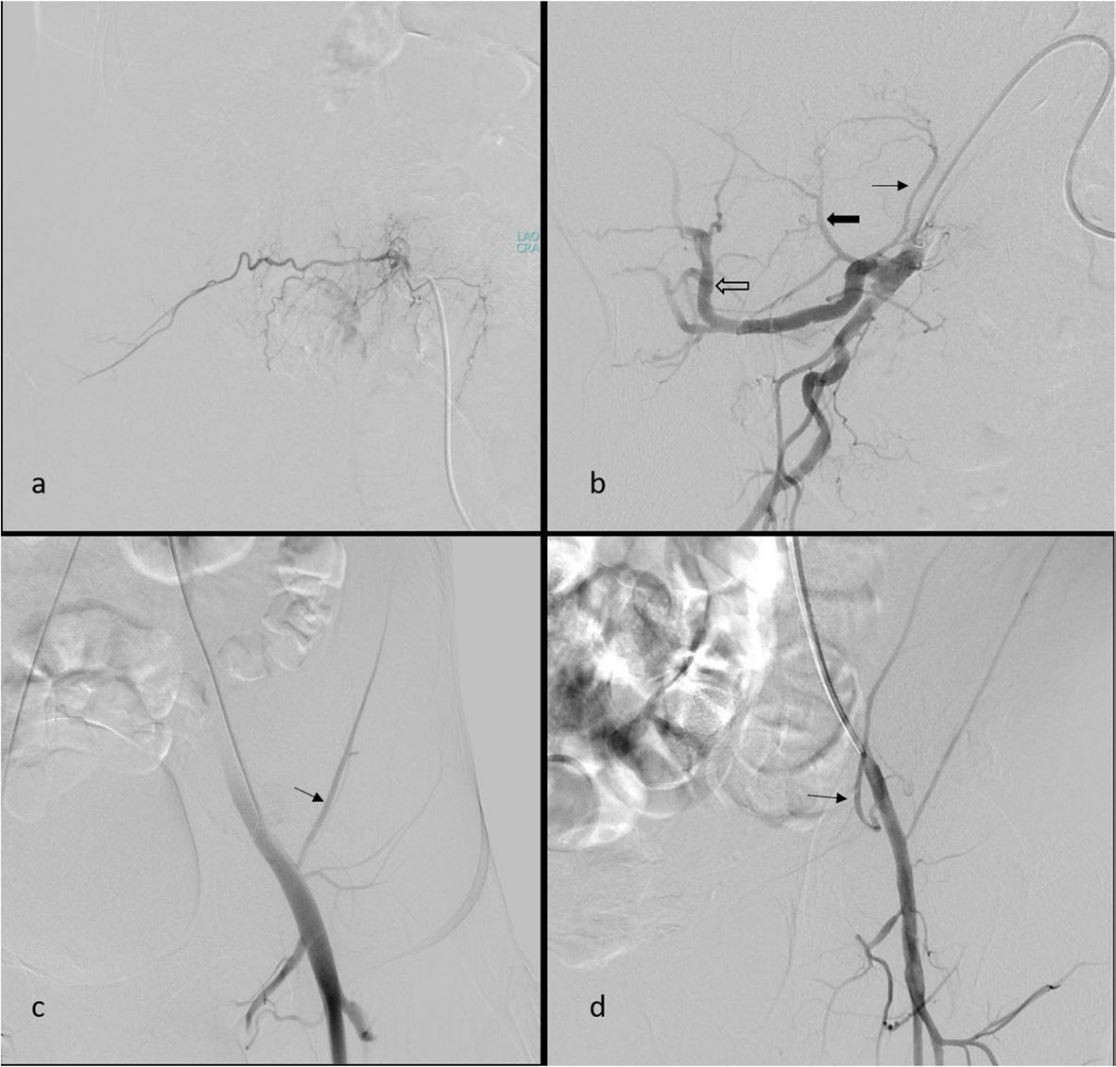
Lumbar artery angiogram obtained from the ostium level using a 4-F vertebral catheter (**a**). Right internal iliac angiogram acquired in a right ipsilateral oblique position, illustrating branches of the posterior division of the internal iliac artery: lateral sacral artery (black arrow), iliolumbar artery (thick black arrow), and superior gluteal artery (hollow black arrow) (**b**). Left external iliac angiograms showing the deep circumflex iliac artery (black arrow) (**c**). Left external iliac angiograms demonstrating the deep inferior epigastric artery (black arrow) (**d**)

**Fig. 6:**
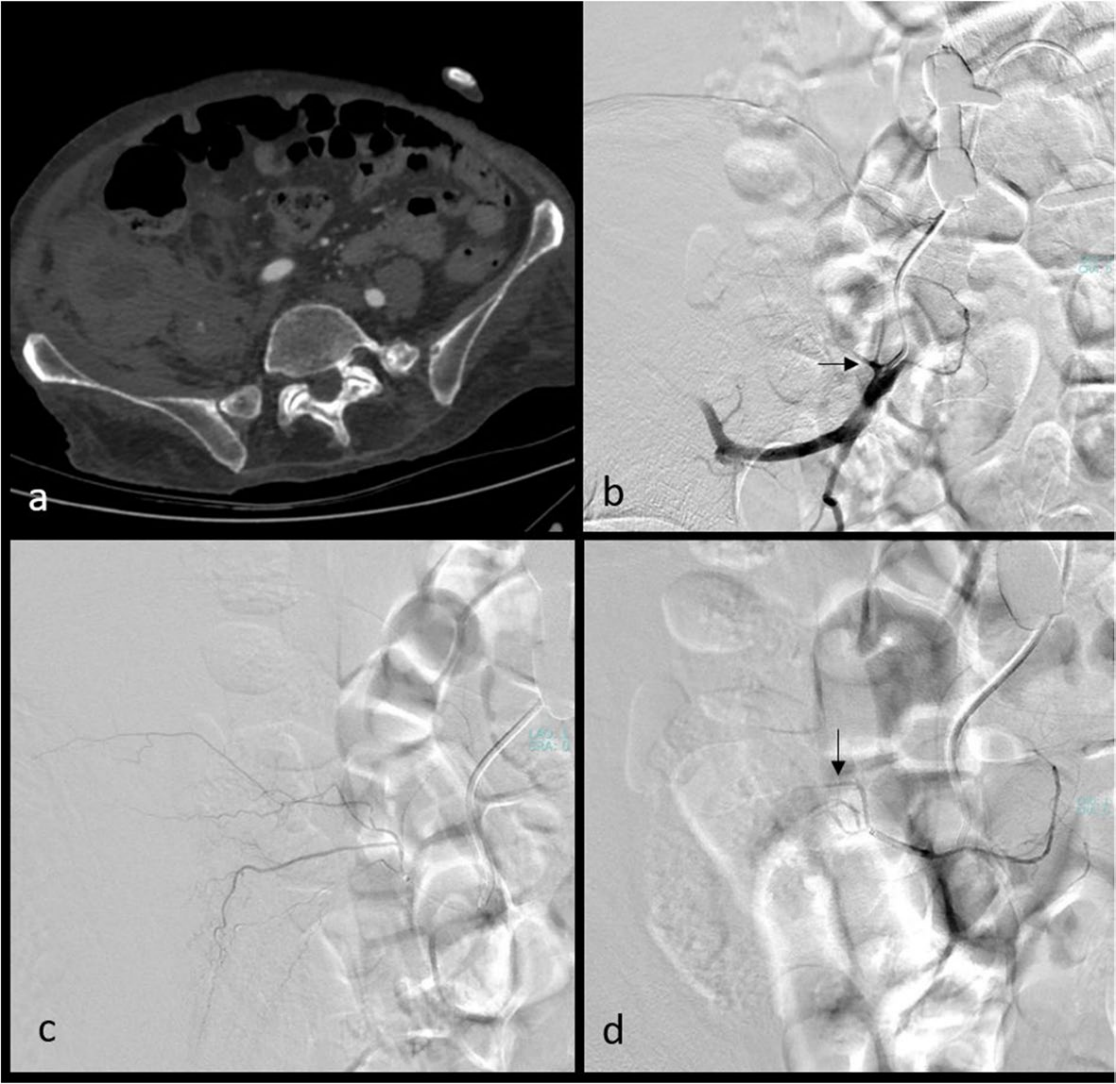
Radiological images of a 58-year-old-male patient with a right iliopsoas hematoma. Computed tomography image (**a**). The right internal iliac arteriogram revealed the iliolumbar artery ostium (black arrow), which was accessed via a coaxial microcatheter (**b**). Despite the absence of active bleeding (**c**), empirical embolization was performed using polyvinyl alcohol particles (250–350 microns) due to the patient’s clinical condition, and complete stagnation of iliolumbar artery flow was achieved (black arrow) (**d**)

**Fig. 7:**
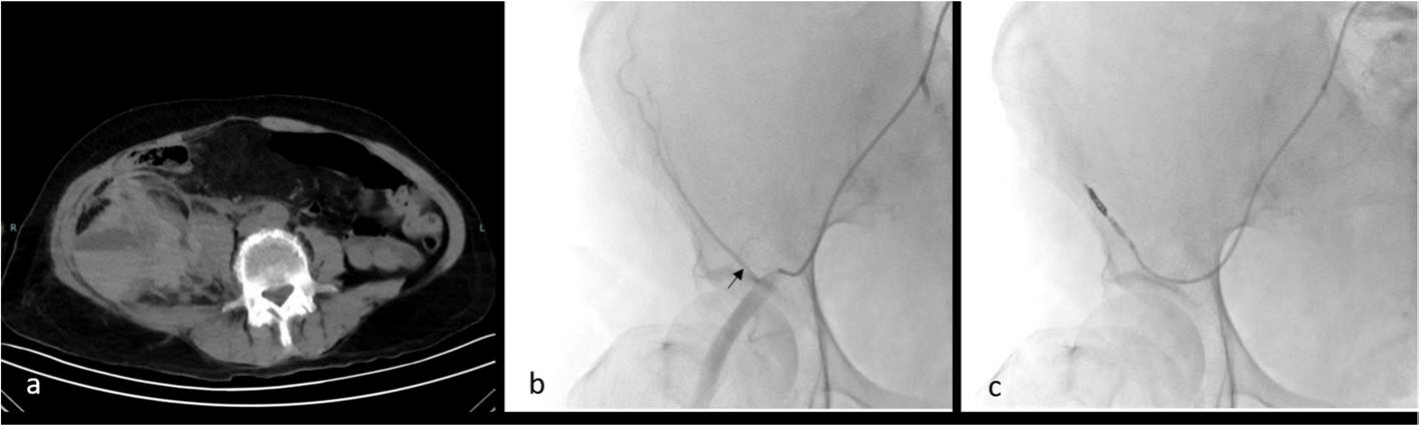
Radiological images of a 73-year-old female patient with a right iliopsoas hematoma. Non-contrast computed tomography image (**a**). The right external iliac arteriogram revealed the ostium of the deep circumflex iliac artery (black arrow), which was accessed via a coaxial microcatheter (**b**). Despite no evidence of active bleeding, empirical embolization was performed using coils due to the patient's clinical condition (**c**)

For PAWH, contralateral femoral access with a Cobra-2 catheter is preferred. In cases where iliac artery tortuosity or an acute aortic bifurcation angle makes crossover with a Cobra-2 catheter unfeasible, a Simmons catheter is preferred for crossing the iliac bifurcation. We sequentially (in a craniocaudal direction) evaluate the LA, the internal iliac artery (IIA) with its posterior division and the ILA, followed by the external iliac artery (EIA), and the DCIA, all ipsilateral to the hematoma (flowchart available in Fig. 4; Fig. 5a–c, Videos 1–3). If active extravasation is identified on lumbar artery angiograms, careful evaluation and embolization, if necessary, of the adjacent lumbar arteries at one level above and below should be considered to prevent persistent bleeding through potential collateral pathways. While advancing from the main trunk of the IIA toward the posterior division, we find it particularly helpful to use ipsilateral oblique imaging in combination with the relatively horizontal lateral course of the superior gluteal artery as a reliable anatomical landmark. When neither angiography nor CBCT identifies primary or secondary signs of bleeding, we recommend empirical embolization of the ILA (a branch of the IIA) and the DCIA (a branch of the EIA), based on the patient’s clinical status and hemorrhage severity, in coordination with the referring clinical team (Figs. 6 and 7). In our institutional practice, this coordination involves real-time discussion with the emergency and intensive care teams to evaluate hemodynamic stability, ongoing transfusion needs, and the absence of alternative bleeding sources before proceeding with embolization.

**Fig. 8:**
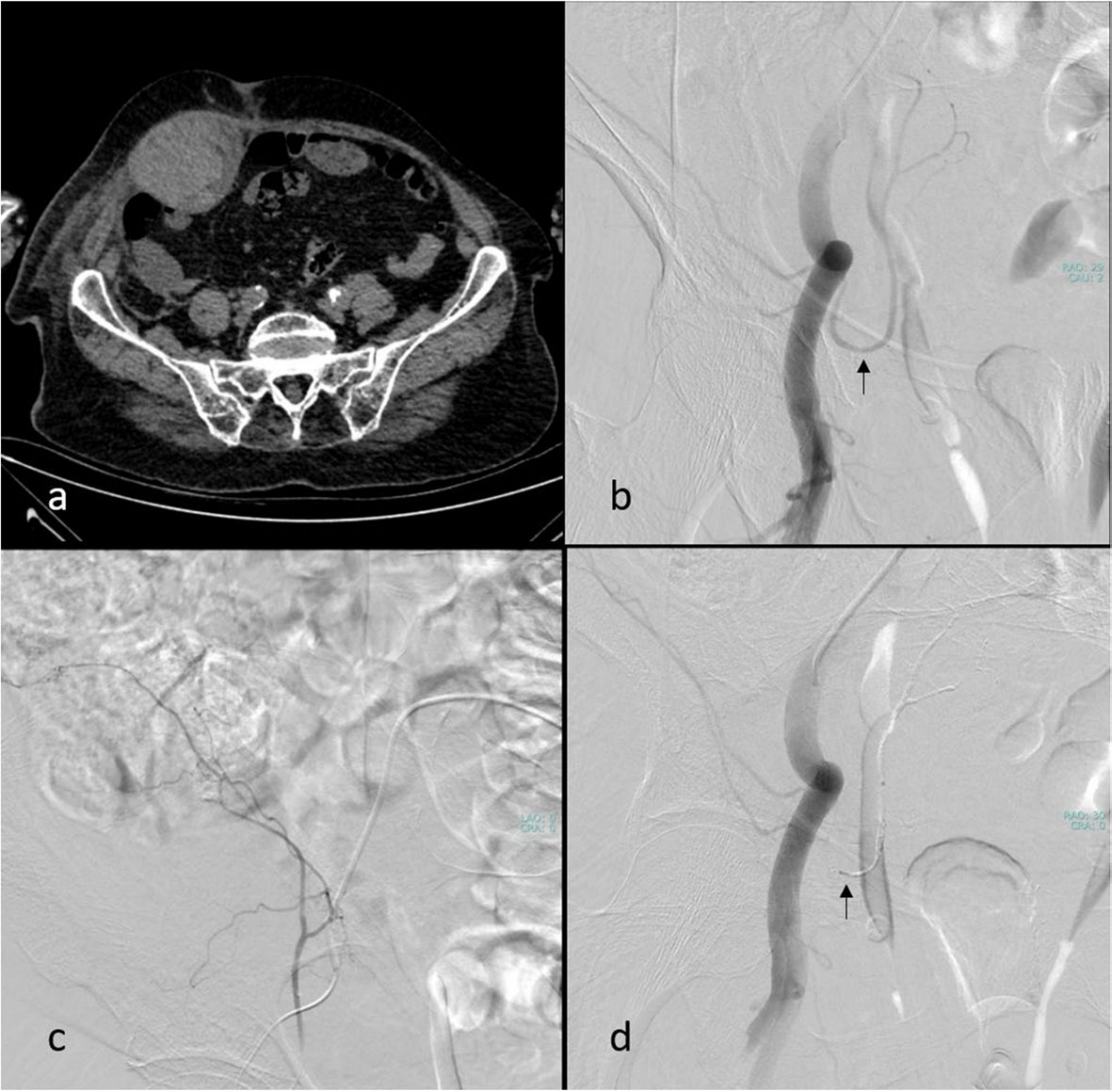
Radiological images of a 77-year-old female patient with a right rectus sheath hematoma. Non-contrast computed tomography image (**a**). The right external iliac angiogram shows the deep inferior epigastric artery (black arrow), which was accessed via a coaxial microcatheter (**b**). Despite the absence of active bleeding on imaging (**c**), embolization was performed using a glue-lipiodol mixture (1:8 concentration) (black arrow) (**d**)

For AAWH, contralateral femoral access is also preferred. After crossing the iliac bifurcation, the DIEA (Fig. 5d, Video 4) is catheterized and embolized, even in the absence of contrast extravasation (Fig. 8). Superior epigastric artery embolization is rarely required and is considered only when CTA demonstrates upper abdominal wall bleeding. Although ipsilateral access with an upward-angled vertebral catheter may seem ideal for DIEA catheterization, given the cranially directed course of the DIEA from the distal EIA, this approach has disadvantages. Specifically, the vascular sheath may be positioned proximally to the DIEA ostium, limiting catheter manipulation. In addition, in rare cases of bleeding from the superficial inferior epigastric artery, a branch of the common femoral artery, ipsilateral access may prevent selective catheterization. This artery is not routinely catheterized unless active extravasation is detected on EIA angiogram.

The proposed stepwise protocol ensures rapid, targeted embolization with minimal equipment change. To the best of our knowledge, no prior study has outlined a procedural strategy specifically for CTA-negative AWH. The approach we propose, rooted in our institutional experience, is intended to address this gap and offer practical guidance for interventionalists managing similar clinical scenarios.

In our experience to date, this strategy has been applied to 54 CTA-negative AWH cases across two centers, achieving 100% technical and clinical success with no instances of rebleeding or in-hospital mortality. Embolization was performed for AAWH in 14 patients and for PAWH in 40 patients, and all procedures were conducted in accordance with the stepwise protocol described for CTA-negative cases. Among AAWH patients, only two demonstrated DSA-positive findings, while the remaining cases (12/14) were DSA-negative and underwent empirical embolization. In all targeted or empirical interventions performed for AAWH, the DIEA was embolized. Similarly, the majority of PAWH cases (37/40) were DSA-negative, and these patients underwent empirical embolization of the ILA and DCIA following the procedural framework outlined in this review. Of the three DSA-positive cases, sequential lumbar arteries were embolized in two, and the iliolumbar artery was embolized in one. The embolic agents used, in order of frequency, were PVA particles (29/54), coils (13/54), and NBCA–lipiodol mixture (12/54).

## Conclusions

Endovascular embolization is an effective and safe treatment for spontaneous AWH. CTA assists in preprocedural planning but may miss bleeding due to intermittent hemorrhage or hemodynamic compromise. DSA remains essential for localization and embolization. Empirical embolization can be safely performed in DSA-negative patients, provided that careful attention is paid to avoid over-embolization and non-target ischemia. In CTA-negative cases, although catheterization of the arteries anatomically supplying the hematoma is recommended for both diagnostic and therapeutic purposes, localization of the bleeding source and achieving effective embolization can remain technically challenging. As no standardized procedural strategy has yet been proposed in the literature for such scenarios, this gap provided the rationale for the procedural approach introduced in the present study.

This narrative review summarizes the literature and presents our procedural strategy for managing patients with undetermined bleeding foci. Our approach proposes a practical roadmap for interventional radiologists performing embolization in both AAWH and PAWH, particularly in CTA-negative cases.

## Study limitations

Our study has several limitations. There is a risk of selection bias due to the inclusion criteria and heterogeneity among the reviewed studies. Although our review provides a detailed analysis of the most recent studies and systematic reviews on the topic, it does not qualify as a systematic review itself. The narrative design precludes quantitative synthesis; conclusions should be interpreted as hypothesis-generating. The procedural protocol proposed at the end of the manuscript for managing CTA-negative AAWH and PAWH is not based on published clinical data; rather, it represents a practical, experience-based approach intended to assist interventional radiologists in similar clinical scenarios. In addition, the proposed procedural strategy lacks external validation, and prospective studies or multicenter registries are warranted to validate empirical embolization protocols, optimize embolic agent selection, and assess the reproducibility and safety of the stepwise approach for CTA-negative cases. Our institutional data is retrospective and limited in size.

## Supplementary Information

Supplementary Material 1. Video 1: Cone-beam computed tomography footage demonstrating the vascular territory of the lumbar artery.

Supplementary Material 2. Video 2: Cone-beam computed tomography footage demonstrating the vascular territory of the iliolumbar artery.

Supplementary Material 3. Video 3: Cone-beam computed tomography footage demonstrating the vascular territory of the deep circumflex iliac artery.

Supplementary Material 4. Video 4: Cone-beam computed tomography footage demonstrating the vascular territory of the deep inferior epigastric artery.

## Data Availability

The datasets used and/or analyzed during the current study are available from the corresponding author on reasonable request.

## Abbreviations

AAWH: Anterior abdominal wall hematoma
AWH: Abdominal wall hematoma
CBCT: Cone-beam computed tomography
CT: Computed tomography
CTA: Computed tomography angiography
DCIA: Deep circumflex iliac artery
DIEA: Deep inferior epigastric artery
DSA: Digital subtraction angiography
EIA: External iliac artery
EVOH: Ethylene-vinyl alcohol
HU: Hounsfield unit
IIA: Internal iliac artery
ILA: Iliolumbar artery
LA: Lumbar artery
NBCA: N-butyl cyanoacrylate
PAWH: Posterior abdominal wall hematoma
PVA: Polyvinyl alcohol
US: Ultrasound
